# Efferocytosis-related gene IL33 predicts prognosis and immune response and mediates proliferation and migration *in vitro* and *in vivo* of breast cancer

**DOI:** 10.3389/fphar.2025.1533571

**Published:** 2025-01-22

**Authors:** Xiao He, Xianjie Cheng, Zhun Zhang, Lanhui Chen, Changjun Xie, Mengjie Tang

**Affiliations:** ^1^ The Second Department of Breast Surgery, The Affiliated Cancer Hospital of Xiangya School of Medicine, Hunan Cancer Hospital, Central South University, Changsha, China; ^2^ Department of Breast and Thyroid, The Xiangya Boai Rehabilitation Hospital, Changsha, China; ^3^ Department of Breast and Thyroid Surgery, The Third Xiangya Hospital, Central South University, Changsha, China; ^4^ Department of Pathology, The Affiliated Cancer Hospital of Xiangya School of Medicine, Hunan Cancer Hospital, Central South University, Changsha, China; ^5^ Department of Oncology, The Second Affiliated Hospital, Hengyang Medical School, University of South China, Hengyang, China

**Keywords:** efferocytosis, breast cancer, IL33, prognosis, immune response

## Abstract

**Background:**

Breast cancer (BRCA) has a high incidence among women, with poor prognosis and high mortality, which is increasing year by year. Efferocytosis is a process of phagocytosis of abnormal cells and is of great value in tumor research. Our study seeks to create a predictive model for BRCA using efferocytosis-related genes (ERGs) to explore the significance of efferocytosis in this disease.

**Methods:**

In this research, Differential analysis, and univariate Cox regression were employed to identify genes linked to prognosis in BRCA patients. Then the BRCA patients were categorized into distinct groups using consensus clustering based on prognosis genes. Survival analysis, PCA, and t-SNE were performed to verify these groups. The enrichment of metabolic pathways within the detected clusters was evaluated using gene set variation analysis (GSVA) and gene set enrichment analysis (GSEA). Additionally, single-sample GSEA (ssGSEA) was used to examine changes in immune infiltration and enrichment. A risk prognostic model was constructed utilizing multivariable Cox regression and Least Absolute Shrinkage and Selection Operator (LASSO) analyses, and subsequently validated its predictive accuracy by stratifying patients according to the median risk score. Ultimately, some crucial independent prognostic genes were pinpointed and their expression, roles, and immune characteristics were explored in both laboratory and live models.

**Results:**

Findings revealed 52 differentially expressed genes (DEGs), of which 21 were significantly linked to BRCA outcomes. These 21 genes were utilized for consensus clustering to categorize BRCA patients into two subtypes. Subtype B was linked to a worse prognosis compared to Subtype A, though both subtypes were distinguishable. The enriched pathways were mainly concentrated in Subtype A and were actively expressed in this group. Following this, a prognostic risk model was constructed using five risk genes, which was proven to possess significant predictive value. A significant link was identified between the immune microenvironment and the risk-associated genes and scores. IL33 was identified as an independent prognostic gene with important research value. Its *in vivo* expression results aligned with the data analysis findings, showing low expression in BRCA. Furthermore, overexpression of IL33 significantly inhibited BRCA growth and motility *in vitro* and *in vivo*, while also enhancing their vulnerability to destruction by activated CD8^+^ T cells.

**Conclusion:**

The ERG-based risk model effectively predicts the prognosis of BRCA patients and shows a strong link with the immune microenvironment. IL33 stands out as a significant prognostic marker, crucial in the onset and advancement of BRCA. This highlights the necessity for additional studies and indicates that IL33 might be a potential target for BRCA treatment.

## 1 Introduction

Breast cancer (BRCA) is the most prevalent cancer among women worldwide and the leading cause of cancer-related mortality in women (Sung et al., 2021). In recent years, due to the continuous improvement of technological means, the mortality rate of BRCA has dropped significantly, but its incidence rate has been slowly increasing (Siegel et al., 2021). Numerous factors influence the incidence of BRCA, including obesity, alcohol consumption, and genetic predispositions, among others (Tamimi et al., 2016; Cathcart-Rake et al., 2018). The most common diagnostic method to confirm BRCA is pathological biopsy. Once determined, biomarker detection is required, because these markers have a predictive effect on the prognosis and treatment of BRCA (Allison, 2021). Therefore, it is necessary to find the diagnosis and biomarkers with predictive value of BRCA.

Efferocytosis is the mechanism through which phagocytes eliminate apoptotic cells. This process is crucial for maintaining tissue homeostasis under normal physiological conditions and for restoring balance after disease (Arandjelovic and Ravichandran, 2015; Bäck et al., 2019). In chronic inflammatory diseases where efferocytosis is impaired, apoptotic cells accumulate due to defective clearance. This buildup of dead cells can result from necrosis and contribute to autoimmunity, tissue damage, and persistent inflammation (Yurdagul et al., 2017; Kawano and Nagata, 2018; Szondy et al., 2014). Molecules and pathways related to the efferocytosis process are closely related to cancer progression, metastasis, and treatment resistance (Tajbakhsh et al., 2021a). Efferocytosis can alter the tumor microenvironment (TME) by inducing immunosuppressive and tolerant conditions through complex signaling pathways. This alteration influences crucial immune mechanisms within tumors, such as the polarization of tumor-associated macrophages (TAMs), T-cell growth, and the secretion of immunosuppressive cytokines. Together, these alterations allow cancer cells to escape immune detection and promote tumor growth (Zhou et al., 2020; Tajbakhsh et al., 2021b; Lei et al., 2020). Studies have found that blocking efferocytosis can improve the function of CD8^+^ cells, thereby reducing pancreatic cancer liver metastasis (Astuti et al., 2024). CD276 can activate lysosomal signaling pathways and transcription factors, thereby enhancing efferocytosis in TAMs, which is crucial in the immunotherapy of bladder cancer (Cheng et al., 2024). In short, the significance of efferocytosis in cancer studies has garnered increasing attention. Although advancements have been made, the exact function of efferocytosis in BRCA is still mostly unknown and not well-defined.

IL33 is a member of the IL-1 cytokine family and is an endogenous alarmin (Schmitz et al., 2005). During inflammation or other types of stress, IL-33 is upregulated and released from necrotic or damaged cells to exert its effects (Rider et al., 2017). In tumors, IL33 has also been found to be involved in the tumor’s pro-oncogenic and anti-oncogenic functions, with its main effects focused on the immune microenvironment, immune occurrence, and tumor-related inflammation (Shen et al., 2018). In head and neck squamous cell carcinoma, cancer-associated fibroblasts were found to release IL-33, which in turn led to migration and invasion through epithelial-mesenchymal transition (Chen et al., 2013). IL-33 is elevated in human BRCA and non-small cell lung cancer (NSCLC) tissues compared to adjacent non-tumor tissues (Liu et al., 2014; Kim et al., 2015). These studies suggest that IL-33 plays a key role in tumors, but research in BRCA is not clear enough.

This study aims to explore the prognostic and immune-related effects of efferocytosis in BRCA through bioinformatics analysis. By validating the role of IL33 both *in vitro* and *in vivo*, the research seeks to clarify how efferocytosis influences BRCA development and progression. The findings could provide new insights and potential treatment strategies for BRCA patients.

## 2 Methods and materials

### 2.1 Data collection

The mRNA sequencing data and clinical details for BRCA individuals were sourced from TCGA (https://cancergenome.nih.gov/), which included 113 normal and 1,118 tumor samples. Initial count data were converted to transcripts per million (TPM), and data were normalized and subsequently transformed to log2 for further analysis. After excluding unusable samples, 1,097 clinical samples were retained for analysis, with detailed clinical information provided in Supplementary Table 1. The GSE58812, GSE21653, and GSE42568 datasets were accessed from GEO (https://www.ncbi.nlm.nih.gov/geo). This dataset underwent both quantile normalization and log2 transformation before being analyzed. A total of 272 ERGs were identified from the GeneCards database (https://www.genecards.org/) and the KEGG database (https://www.kegg.jp/entry/hsa04148), as listed in Supplementary Table 2.

### 2.2 Differential gene identification and mutation frequency analysis

The “limma” package was utilized to identify DEGs between healthy and cancerous samples, applying thresholds of |logFC| > 1 and a *p*-value <0.05. The “pheatmap” package was employed to generate a heatmap illustrating the DEGs. Following this, a univariate Cox regression analysis was conducted to pinpoint genes linked to prognosis. Finally, the “RCircos” package in R was used to map copy number variation (CNV) alterations of prognosis-related genes across 23 chromosomes.

### 2.3 Consistent clustering analysis of ERGs

The BRCA dataset was analyzed to identify unsupervised subgroups and clusters based on DEGs using the R package “ConsensusClusterPlus”. The authenticity of these clusters was additionally verified using principal component analysis (PCA), t-distributed stochastic neighbor embedding (t-SNE), and uniform manifold approximation and projection (UMAP), with the help of the R packages “ggplot2″, “Rtsne”, and “ump”, respectively. The Kaplan-Meier (K-M) survival plots for each subgroup and cluster were examined and displayed utilizing the “survival” and “survminer” packages in R.

### 2.4 GSVA and functional enrichment analysis of ERGs

We employed the R package “GSVA” alongside the KEGG pathway database to explore biological mechanisms across various subgroups. We applied the ssGSEA algorithm to investigate the association between immune cell infiltration and the various subgroups. Immune cell infiltration outcomes were depicted with the R package “ggplot2”.

### 2.5 Construction and validation of prognostic model of ERGs

BRCA patients were split into training and test groups in equal proportions through random assignment. We employed the LASSO Cox regression method to identify possible prognostic indicators and create ERG prognostic scoring models. The risk score of the model is derived by calculating the expression levels of selected genes in conjunction with their respective regression coefficients, utilizing the fundamental formula: Prognostic Risk Score = $\sum_{i=1}^{n}\exp-genei*coef-genei$ . During the evaluation, individuals from both the training and testing datasets were divided into high-risk and low-risk categories according to the median risk score. To assess variations in survival, K-M survival plots were created with the R package “survival” and overall survival (OS) was contrasted between the two cohorts. We evaluated the model’s effectiveness over time by generating ROC curves at 1, 3, and 5-year intervals using the “timeROC” package in R. Additionally, a nomogram for predicting overall survival was constructed using the R package “rms”, incorporating risk scores along with clinicopathological characteristics such as age, sex, and stage. Visualization of cluster distributions and survival outcomes was accomplished using Sankey plots created with the R packages “dplyr”, “ggplot2″ and “ggalluvial”. Ultimately, the dependability and efficiency of the nomograms were assessed using various measures, such as the concordance index (C-index), calibration plots, and decision curve analysis (DCA) (Kerr et al., 2016; Vickers et al., 2016).

### 2.6 Analysis of drug response and immune cell presence

The CIBERSORT algorithm was applied to explore the associations between prognostic genes, risk scores, and the immune cell populations infiltrating tumors. To assess the stromal, immune, and ESTIMATE scores for BRCA, we utilized the R package “estimate”. The CIBERSORT R script v1.03 was utilized to approximate the ratios of 22 distinct immune cell types.

Furthermore, to explore BRCA’s responsiveness to different anti-cancer medications, we utilized the Genomics of Drug Sensitivity in Cancer (GDSC) portal at https://www.cancerrxgene.org/ (accessed on 18 March 2024) for drug profile information (Yang et al., 2013). The “pRRophetic” R package was utilized to determine the half-inhibitory concentration (IC50) values, which were then used to assess drug sensitivity among various samples.

### 2.7 Screening of independent prognostic genes with research value

The research identified crucial predictive genes through the application of both univariate and multivariate Cox regression methods. The “forestplot” package was utilized to create visual depictions of the results, illustrating *p*-values, hazard ratios (HR), and 95% confidence intervals (CI) for each gene. The expression levels of these genes in both BRCA and normal tissues were illustrated utilizing the “ggplot2″ package. Survival differences between groups with elevated and reduced gene expression were assessed using K-M survival analysis and the Log-rank test. The K-M plots offered *p*-values and HR (95% CIs) to evaluate survival outcomes according to the expression levels of prognostic genes.

### 2.8 Statistical analysis

PERL (v5.30.0) was utilized to annotate and curate transcriptome, clinical, and gene expression information. Data analysis was conducted using R software (v4.3.3), with a *p*-value under 0.05 deemed significant.

### 2.9 Tissue samples

From January to June 2024, twelve pairs of BRCA and adjacent normal tissues were gathered from the Affiliated Cancer Hospital of Xiangya School of Medicine of Central South University. These samples are primarily used for verifying IL33.

### 2.10 Immunohistochemical staining

The adjacent and BRCA tissue samples were preserved in 4% paraformaldehyde, embedded in paraffin, and cut into 6 μm thick slices. These slices underwent a process of dewaxing and rehydration to prepare them for immunohistochemical staining. First, the paraffin-embedded tissue sections were dewaxed and rehydrated to remove the paraffin and restore hydration. Next, to unmask the antigens and block endogenous peroxidase activity, the rehydrated tissue sections were treated with a Tris-EDTA buffer solution containing 10 mM Tris-HCl and 1 mM EDTA. The sections then underwent heat-induced epitope retrieval by being heated in a pressure cooker for 5 min until boiling. After this, the sections were washed three times to eliminate any residual buffer. Following the washes, the tissue sections were incubated with a blocking solution, such as bovine serum albumin (BSA), for 30 min to prevent non-specific binding. Subsequently, the sections were incubated overnight at 4°C with the primary antibody IL33 to facilitate specific antigen-antibody binding. The next day, the tissue sections were incubated with a secondary antibody for 1 h at room temperature to amplify the signal generated by the primary antibody. To visualize the nuclei, the sections were counterstained with hematoxylin. Finally, the tissue sections were dehydrated, mounted with a neutral resin, and coverslipped for microscopic examination. This detailed protocol ensured proper antigen retrieval, specific antibody binding, and visualization of the target protein in the tissue samples, allowing for accurate immunohistochemical analysis of IL33 expression in both the adjacent normal and BRCA tissues.

### 2.11 Quantitative real-time PCR

The process of total RNA extraction, cDNA synthesis, and PCR amplification for gene expression analysis involved several detailed steps to ensure accurate and reliable results. We extracted total RNA using the RNeasy Mini Kit (QIAGEN, Beijing, China). The concentration and purity of RNA were determined using a Nanodrop luminometer (an A260/A280 ratio between 1.8 and 2.0 is generally considered pure). cDNA synthesis was carried out with a high-capacity cDNA reverse transcription kit (Thermo, Shanghai, China). PCR was conducted using TaqMan Gene Expression Master Mix (Bio-Rad, Shanghai, China) according to the manufacturer’s instructions, with TaqMan probes for human IL33 and actin obtained from Sangong Biotechnology (Shanghai, China).

### 2.12 Western blot

Cells were transfected with a lentivirus carrying either an IL33 overexpression plasmid (OE) or a control plasmid (OC) for 48 h. Following transfection, proteins were isolated and analyzed using SDS-PAGE. Subsequently, the proteins were moved onto a PVDF membrane. Detection of the antigen-antibody interaction was achieved using an IL33 primary antibody for 16–18 h. After the membranes were washed by TBST 5 times, this interaction was visualized using a secondary antibody linked to peroxide and the ChemiDoc system. The strength of the bands was quantified with ImageJ software.

### 2.13 MTT

Cells were infected with lentivirus containing IL33 overexpression vector or control vector. After 1 day of infection, cells were seeded in 96-well plates at a concentration of 6,000 cells per well. After cell seeding, they were allowed to fully adhere to the wall and recover to normal growth conditions. Next, 50 μM MTT solution (concentration of 2 mg/mL) was added to each well and incubated for another 4 h so that MTT was reduced by mitochondria in the cells to form purple Formazan crystals. After the incubation, the medium in each well was carefully discarded, and the cells were washed twice with PBS to remove the unreduced MTT solution. Subsequently, 150 μL of DMSO was added to each well to fully dissolve the Formazan crystals. To ensure complete dissolution, each well was treated with gentle stirring or shaking for 10 min. Finally, the absorbance (OD value) of each well was measured at a wavelength of 490 nm using a microplate reader, which reflects the number of surviving cells. The absorbance value is positively correlated with the degree of cell survival and can reflect the effect of IL33 overexpression on cell growth or proliferation.

### 2.14 Clonogenic assay

Cells were infected with lentivirus containing IL33 overexpression plasmid or control plasmid for 24 h, then placed in 24-well plates, 1,000 cells per well, and stored in 10% formaldehyde after 6–8 days, usually incubated for 10–15 min to ensure that the cells are fully fixed. After fixation, the formaldehyde solution was discarded, and the cells were washed 2–3 times with PBS to remove excess formaldehyde. Then they were stained with 0.1% crystal violet and incubated at room temperature for 10–20 min. After staining, the cells were washed again with PBS to remove unbound crystal violet. The absorbance was measured at 550 nm.

### 2.15 Scratch assay

Cells were infected with a lentivirus containing either an IL33 overexpression plasmid or a control plasmid for a period of 24 h. Post-transfection, the cells were seeded into a 12-well plate at a density of 3×10^5^ cells per well. After an additional 24 h, a scratch was introduced into the cell monolayer using a 200 μL pipette tip. The cells were subsequently washed twice with phosphate-buffered saline and incubated in serum-free medium for a defined period. Wound images were captured using a microscope at 0 h and 24 h post-scratch. The width and area of the scratches were quantified using ImageJ software.

### 2.16 Transwell assay

For a duration of 24 h, cells were infected with a lentivirus harboring either an IL33 overexpression plasmid or a control plasmid. Post-transfection, 40,000 cells suspended in 200 μL of serum-free medium were seeded into the upper chamber of a Transwell apparatus, while 600 μL of medium containing 10% serum was utilized as a chemoattractant in the lower chamber. After an additional 24-h incubation period, the cells were fixed with 4% paraformaldehyde and subsequently stained with 0.1% crystal violet. Non-invasive cells located on the apical surface of the membrane were carefully removed using a cotton swab, and the residual cells were subsequently imaged using microscopy.

### 2.17 *In vivo* tumor models

Female BALB/c nude mice, 5–6 weeks old, were obtained from Skajingda Biotechnology and kept in specific pathogen-free conditions. The environment was maintained at 22°C–26°C, with about 55% humidity and a 12-h light/dark cycle. Mice, five per cage, were randomly assigned to different experimental groups to minimize bias. For xenograft modeling, they were subcutaneously injected with 2×10^6^ MDA-MB-231-OC or MDA-MB-231-OE cells. Tumor size was calculated using formula 1/2 × (long diameter × short diameter^2^), and mouse weight was recorded every other day once tumors reached 70–100 cm^3^. All procedures adhered to China’s regulations for laboratory animal use and care.

### 2.18 CD8^+^ T cell cytotoxic assay

CD8^+^ T cells were isolated following established protocols from the literature (Shen et al., 2022). Tumor cells were transfected with lentivirus-packaged overexpressed IL33 plasmid or control plasmid and incubated for 24 h. Subsequently, the cells were placed into a 96-well plate, with each well containing 10,000 cells. CD8^+^ T cells were introduced into the wells following 12 h. The culture was incubated for 2 days, after which the medium was discarded, and the tumor cells were rinsed twice with PBS to eliminate the T cells. Subsequently, the MTT test was conducted to evaluate the cytotoxic impact of CD8^+^ T lymphocytes on cancer cells.

## 3 Results

### 3.1 Validation and mutation frequency analysis of differentially expressed genes in BRCA

**FIGURE 1:**
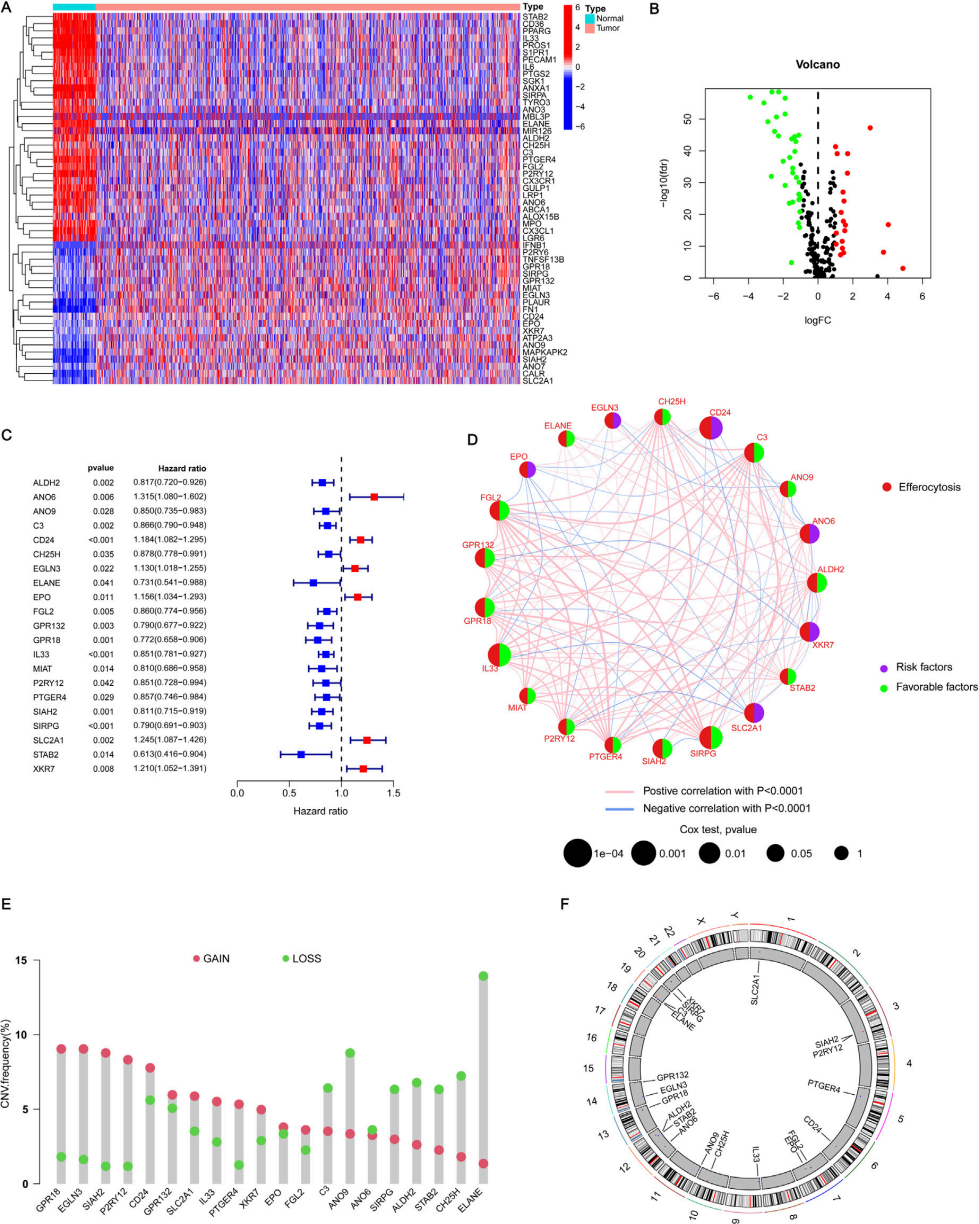
Acquisition of ERGs and CNV analysis. **(A)** The heatmap illustrates the expression levels of DEGs in both BRCA and normal tissues. In this visualization, red signifies a positive correlation, indicating elevated expression levels, whereas blue represents a negative correlation, denoting reduced expression levels. **(B)** The volcano plot delineates the ERGs that satisfy the criteria of |logFC| ≥ 1 and a *p*-value <0.05. Genes are graphically represented based on their fold change and statistical significance, with those meeting the specified thresholds distinctly marked. **(C)** The forest plot illustrates the 21 most significant differentially expressed genes (DEGs) identified through univariate Cox regression analysis, each exhibiting a *p*-value <0.05. This plot visually depicts the hazard ratios (HR) and their corresponding 95% confidence intervals (CI) for each gene, thereby underscoring their prognostic relevance. **(D)** The network diagram depicts the relationships among the top 21 DEGs. This visualization shows how these genes interact or correlate with each other, providing insights into their collective role in BRCA and their potential interactions within the biological network. **(E)** In the visualization, red spots represent areas where copy number amplification is frequent (gain) for each ERG, while blue spots indicate regions with copy number deletions (loss) for these genes. **(F)** The CNV cycle diagram illustrates the distribution and CNVs of ERGs across different chromosomes. This diagram shows where ERGs are located on the chromosomes and whether there are amplifications or deletions in their copy numbers.

In our study, we investigated the differential expression of ERGs in BRCA on the TCGA-BRCA cohort. Our analysis revealed 52 genes with differential expression, out of which 32 genes showed higher expression in normal tissues and 20 genes exhibited higher expression in BRCA (Figure 1A). Figure 1B illustrates a volcano plot that shows gene expression, where green indicates genes that are downregulated and red signifies those that are upregulated. These identified genes are referred to as differentially expressed genes (DEGs). Then we verified the GO enrichment of these 32 DEGs. GO includes three parts: BP (Biological Process), CC (Cellular Component), and MF (Molecular Function). In BP, it is mainly enriched in pathways such as LOCOMOTION and CELL_MIGRATION (Supplementary Figure S1A). In CC, it is mainly enriched in pathways such as CELL_SURFACE and INTRINSIC_COMPONENT_OF_PLASMA_MEMBRANE (Supplementary Figure S1B). In MF, it is mainly enriched in pathways such as SIGNALING_RECEPTOR_BINDING and MOLECULAR_TRANSDUCER_ACTIVITY (Supplementary Figure S1C). This insight allows us to have a deeper understanding of the biological functions and potential impacts of these DEGs in the context of the study. To enhance the ERG signature, we conducted a univariate Cox regression analysis on these DEGs and discovered 21 key genes linked to BRCA prognosis. Among these, 15 genes were categorized as low-risk (labeled in blue, HR < 1), and six genes were classified as high-risk (labeled in red, HR > 1) (Figure 1C). The correlation between these genes is illustrated in Figure 1D, showing predominantly positive correlations. These discoveries underscored the crucial significance of these genes in predicting BRCA and warrant further investigation.

Subsequently, we analyzed the copy number variation (CNV) status of prognosis genes, recognizing the potential impact of CNV on BRCA development and progression. Our results indicated that 13 genes, including CPR18 and IL33, exhibited a higher frequency of copy number amplification compared to copy number deletion, while eight genes, including C3 and ANO9, demonstrated a lower frequency of copy number amplification than copy number deletion (Figure 1E). Furthermore, the circular plot illustrating copy numbers showed the mutation sites of these 21 genes across 23 chromosome pairs, revealing mutations on 13 of them (Figure 1F). These mutation results are crucial for elucidating the pathogenesis of BRCA and identifying potential therapeutic targets.

### 3.2 Consensus clustering identifies two subtypes of BRCA

**FIGURE 2:**
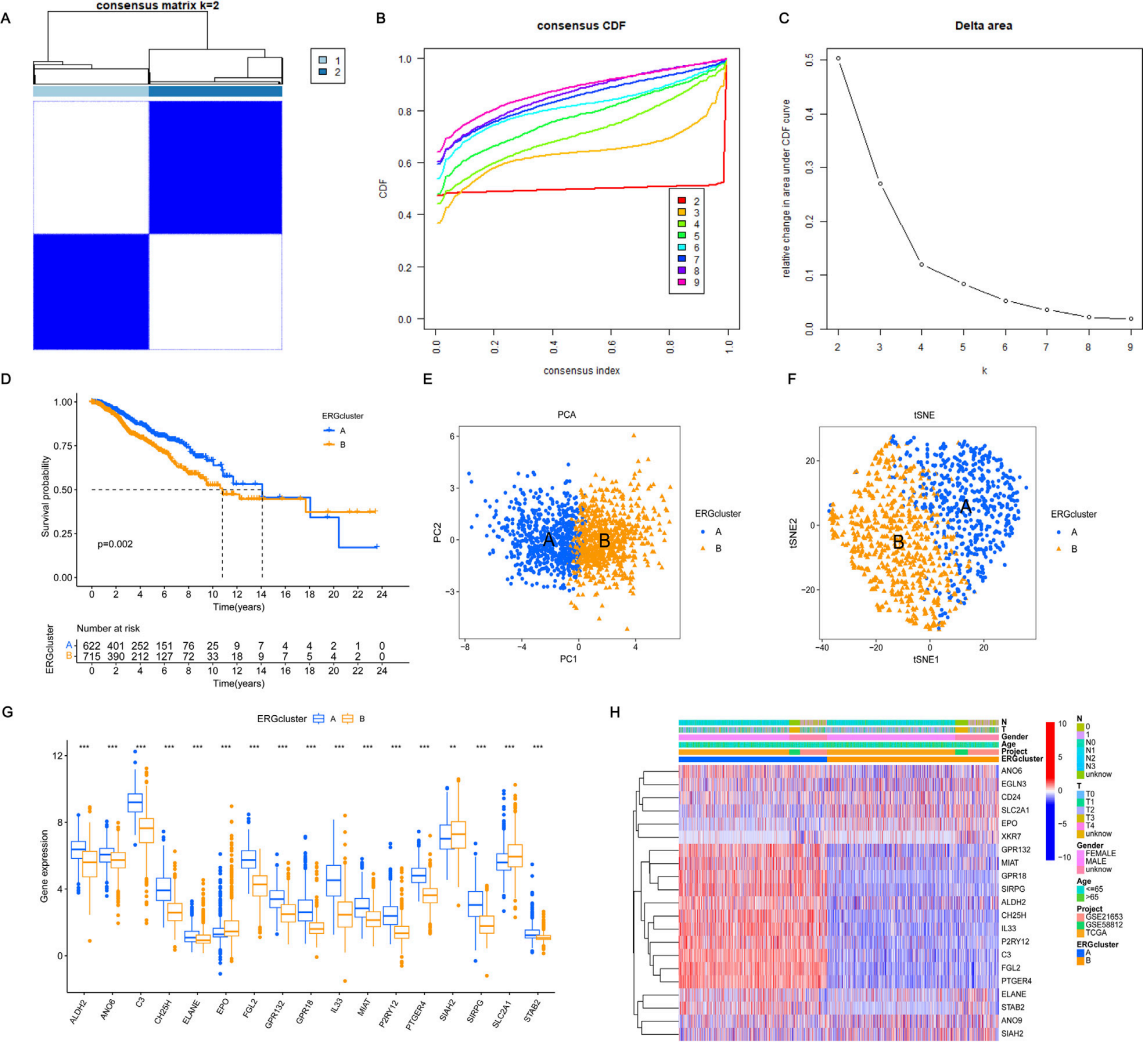
Consensus clustering and validation based on 21 key DEGs. **(A–C)** Consensus clustering of the 21 significant DEGs revealed that the optimal number of clusters is k = 2, effectively categorizing BRCA patients into two distinct groups. **(D)** The survival probability for patients in groups A and B varies with survival events, reflecting differences in their overall survival rates over time. **(E, F)** Principal Component Analysis (PCA) and t-distributed Stochastic Neighbor Embedding (t-SNE) were performed to differentiate between the two clusters. In the visualizations, blue denotes cluster A, and yellow denotes cluster **(B)** A clearer separation between the two clusters indicates a more accurate grouping based on the 21 significant DEGs. **(G)** The expression differences of these DEGs between the two groups were analyzed to assess how the levels of these genes vary between cluster A and cluster B. **(H)** The expression of these DEGs between the two subgroups was evaluated, taking into account clinical characteristics such as age, gender, and T and N stages. Statistical significance was determined with the following thresholds: *p < 0.05, **p < 0.01, and ***p < 0.001.

Performing unsupervised consensus clustering analysis is crucial for identifying subtypes within different subgroups. In this study, we used consensus cluster analysis based on 21 important ERGs and found that K = 2 was the best classification for BRCA. This division resulted in two patient groups (Figures 2A–C). Analyzing the survival difference between these two groups revealed that both groups experienced a decrease in survival probability over time, with group A exhibiting a higher median survival time than group B. Figure 2D illustrated a notable disparity in the likelihood of survival between the two cohorts. Both PCA and tSNE analyses were able to differentiate between the two patient groups, suggesting that these genes could effectively classify the two subtypes (Figures 2E, F). Further analysis of the gene expression of these 21 genes between the two subtypes showed that 17 genes exhibited significant differences. Interestingly, 14 genes including ALDH2 and ANO6 showed significant expression in cluster A. Furthermore, three genes, including SLC2A1, EPO, and SIAH2, exhibited high expression levels in cluster B (Figure 2G). Upon combining these findings with the clinical characteristics of BRCA, the heatmap illustrating gene expression between the two subtypes also revealed differences in expression (Figure 2H). These results demonstrated that the characteristics based on these 21 genes can effectively distinguish different clusters and offer the potential to provide diverse treatment strategies to optimize treatment methods for patients with varying types of BRCA.

### 3.3 Identification of enriched pathways and immune preliminary validation of different subtypes

**FIGURE 3:**
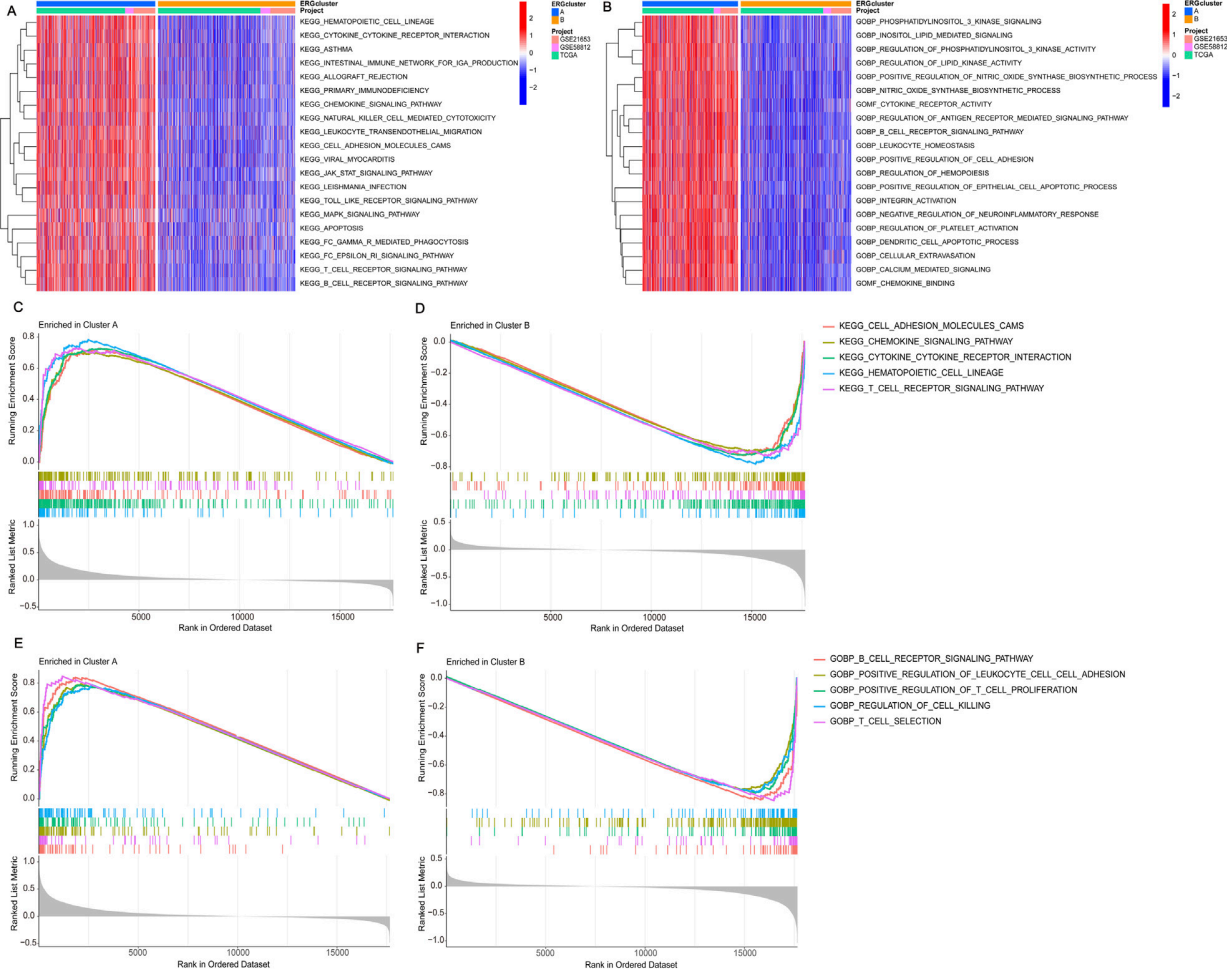
Performing gene set enrichment analysis. **(A)** The KEGG enrichment analysis of DEGs highlighted potential mechanisms associated with the two DEG patterns. **(B)** The GO enrichment analysis of DEGs revealed potential mechanisms underlying the two identified DEG patterns. **(C, D)** Gene Set Enrichment Analysis (GSEA) revealed that the top five KEGG signaling pathways are most active in cluster A. **(E, F)** GSEA also showed that the top five GO signaling pathways are highly active in cluster A.

Identifying differences in pathway expression in patients with different clustering types of BRCA can potentially help us understand the development of BRCA and provide potential pathways for treatment. To achieve this, we conducted KEGG and GO pathway validation for the dichotomous classification. Among the 20 pathways identified, they are mainly enriched in cluster A, regardless of KEGG or GO enrichment analysis (Figures 3A, B). Validation of KEGG enriched pathways by GSEA revealed that CELL_ADHESION_MOLECULES_CAMS, CHEMOKINE_SIGNALING_PATHWAY, CYTOKINE_CYTOKINE_RECEPTOR_INTERACTION, HEMATOPOIETIC_CELL_LINEAGE, and T_CELL_RECEPTOR_SIGNALING_PATHWAY were all enriched in cluster A (Figures 3C, D). The verification of GO pathway enrichment also indicated that B_CELL_RECEPTOR_SIGNALING_PATHWAY, POSITIVE_REGULATION_OF_LEUKOCYTE_CELL_CELL_ADHESION, POSITIVE_REGULATION_OF_T_CELL_PROLIFERATION, REGULATION_OF_CELL_KILLING, and T_CELL_SELECTION were enriched in cluster A (Figures 3E,F). These routes are intimately connected to the onset and progression of BRCA. Hence, identifying their enrichment in different clusters may aid in explaining why different clusters have different survival statuses and finding practical and important targets for the treatment of BRCA. In the end, we analyzed the infiltration of immune cells across the two different subtypes. Using ssGSEA, we discovered notable variations in the infiltration of 23 immune cells between the two subgroups. Cluster A exhibited a high infiltration of most immune cells, whereas cluster B showed significant infiltration predominantly by CD56dim natural killer cells (Supplementary Figure S2). Differences in the extent of immune cell infiltration might explain the survival disparity between the two subtypes.

### 3.4 Build an accurate prognostic risk model

**FIGURE 4:**
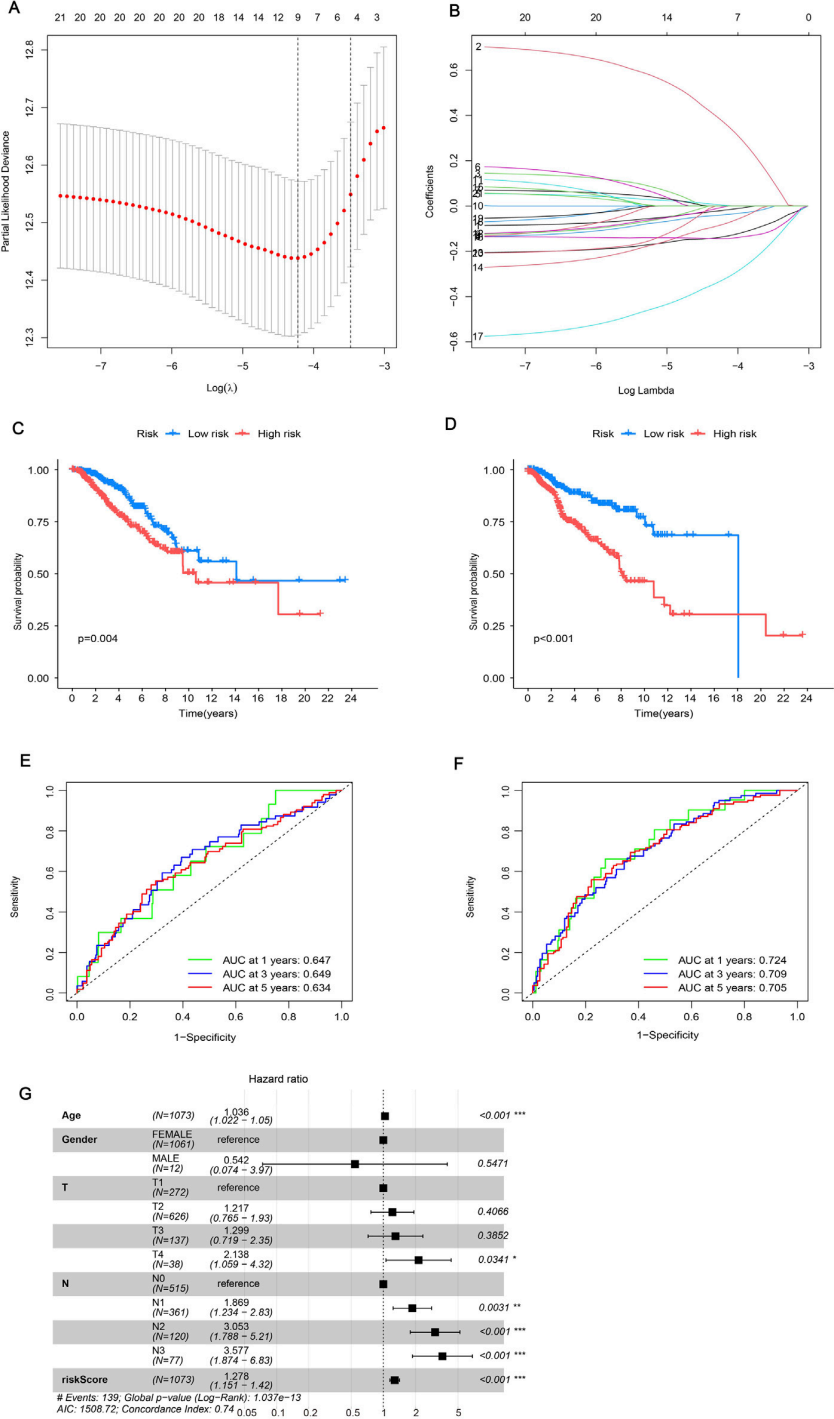
Constructing a prognostic model. **(A, B)** LASSO regression analysis was employed to determine the optimal coefficients and lambda values for the prognostic DEGs, thereby enhancing the model’s predictive accuracy. **(C, D)** Kaplan-Meier (K-M) survival curves were utilized to illustrate variations in prognosis among different risk groups: **(C)** representing the test group and **(D)** representing the training group. **(E, F)** Time-dependent Receiver Operating Characteristic (ROC) curves for Overall Survival (OS) were evaluated at 1-year, 3-year, and 5-year intervals: **(E)** for the test group and **(F)** for the training group. **(G)** Univariate Cox regression analysis was established on all clinical features included in the risk model, with a specified significance threshold set at *p* < 0.05.

In order to assess the impact of ERGs on BRCA, we developed a prognostic risk model. We initially employed LASSO regression to analyze 21 important genes. After addressing multicollinearity and model overfitting, we identified five genes (ANO6, IL33, MIAT, SIAH2, SIRPG) for the risk model (Figures 4A, B). Subsequently, we calculated the risk score formula as follows: risk score = ANO6×0.637634391712056 - IL33 × 0.220865917940123 - MIAT×0.223869462386534 - SIAH2×0.496867864577252 - SIRPG×0.224643319313097, seeing Supplementary Table 2. The study included BRCA patients who were randomly assigned to a training group and a testing group. Subsequently, each cohort was categorized into high-risk and low-risk segments according to their risk scores. Examining the survival disparities between high- and low-risk segments revealed a decline in survival probability over time, with the high-risk group exhibiting notably lower survival rates and shorter median survival duration compared to the low-risk group. This pattern was consistent in both the test and training groups (Figures 4C, D). The subsequent ROC curve analysis revealed that the model constructed was effective in predicting patient survival time, as indicated by AUC values greater than 0.6 (Figures 4E, F). Additionally, an independent prognostic analysis combining the risk score with clinicopathological characteristics found that age, N stage, and risk score were all related to patient prognosis (Figure 4G). This indicated that the survival time of patients can be precisely forecasted using the risk score derived from these genes. The risk heatmap indicated that, apart from ANO6, the remaining genes were low-risk genes (Supplementary Figure S3A). Additionally, examining the relationship between gene clusters and risk models revealed a notable disparity in risk scores, with cluster B exhibiting a marginally higher risk score compared to cluster A (Supplementary Figure S3B). Individuals with elevated risk scores tended to be part of cluster B and progress to high-risk status, whereas those with lower risk scores were more often associated with cluster A and remained low-risk (Supplementary Figure S3C). This could be linked to the varying enrichment outcomes of the two clusters across distinct pathways. Analysis of the risk group revealed that only a small number of patients in this group died, indicating an improved cure rate for BRCA patients.

**FIGURE 5:**
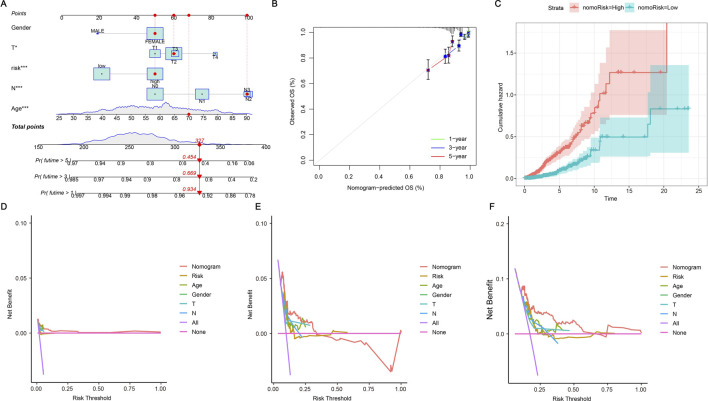
Nomogram further validates risk model. **(A)** A nomogram plot has been developed that integrates ERG scores with clinicopathological features. This tool facilitates the prediction of individual patient outcomes based on their specific scores and characteristics. **(B)** A calibration plot is employed to validate the nomogram by comparing the predicted probabilities of outcomes with the actual observed outcomes. It visually assesses the accuracy of the nomogram’s predictions against real data, with the plot typically featuring a 45-degree line representing perfect agreement. The closer the plotted points are to this line, the more accurate the predictions made by the nomogram. **(C)** The cumulative hazard curve illustrates the probability of survival over time by showing the cumulative risk of experiencing the event as time progresses. The curve typically slopes upward, indicating an increasing hazard over time, and helps in assessing how different factors or interventions affect survival chances. **(D–F)** The Decision Curve Analysis (DCA) curves illustrate the predicted survival rates for BRCA patients at 1, 3, and 5 years as derived from the nomogram. These curves are utilized to assess the clinical utility of the prognostic model by demonstrating the net benefit of using the model at different threshold probabilities.

We confirmed the situation by using risk scores and patients’ clinicopathological characteristics to create a nomogram. Each of the patient’s characteristics corresponds to a score, and their overall score determines their predicted score. This score corresponds to the survival probability at different time points. As shown in Figure 5A, the patient achieved a total of 327 points, indicating survival chances of 93.4% at 1 year, 66.9% at 3 years, and 45.4% at 5 years. Figure 5B shows that the patient’s typical curve is very similar to the optimal curve, suggesting that the model predicts accurately. The likelihood of risk escalated over time, with individuals in the high-risk category encountering more danger compared to those in the low-risk category (Figure 5C). The C-index graph demonstrated that the developed risk model was the most accurate in forecasting survival when compared to clinical factors such as age, gender, and stage (Figures 5D–F). This indicates that our model has strong predictive value for assessing the prognosis and survival probability of BRCA patients.

### 3.5 Immune microenvironment analysis

**FIGURE 6:**
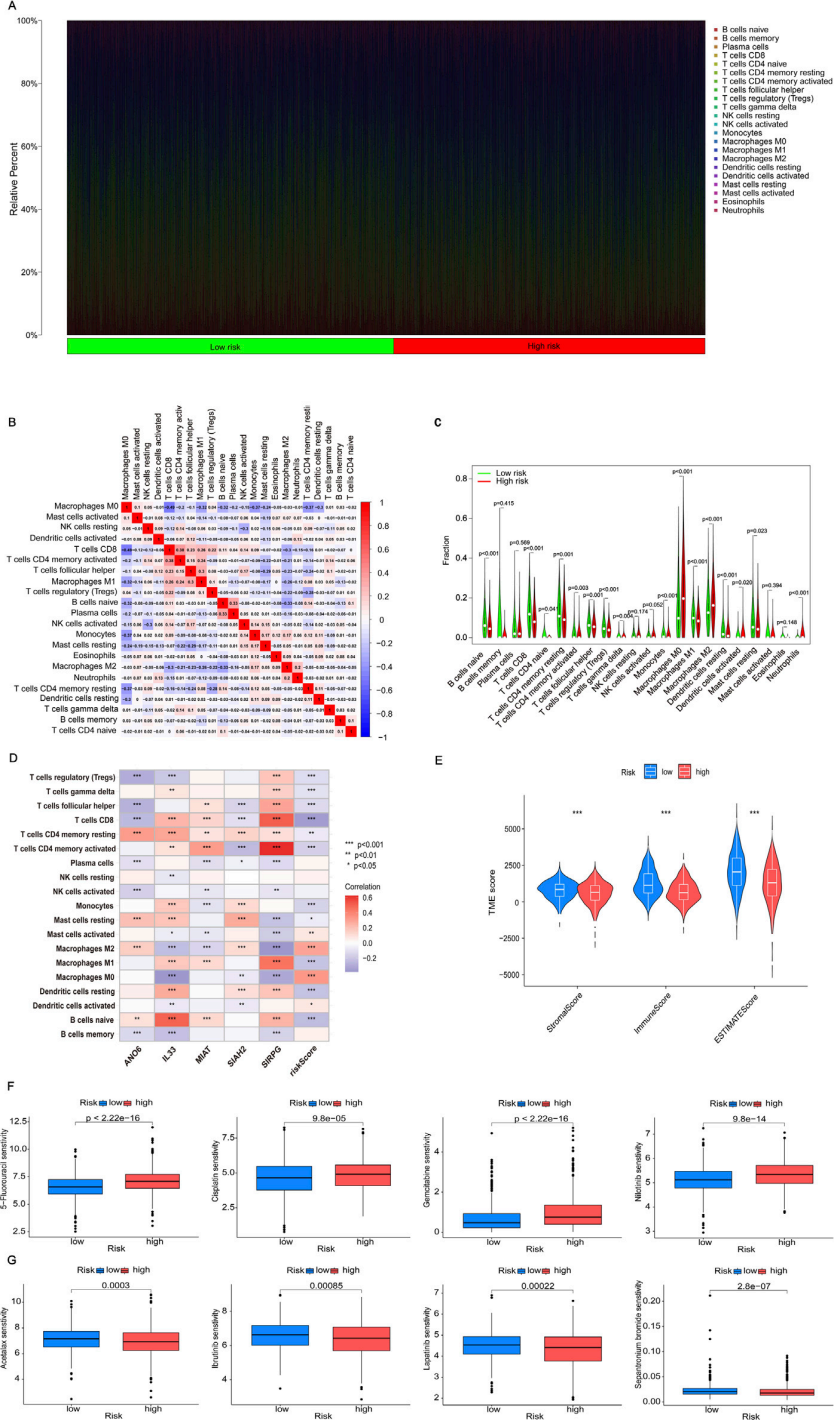
Analyzing immune cell infiltration, the TME and drug sensitivity analysis. **(A)** The proportion of immune cells in each patient is analyzed. **(B)** The correlation between immune cell populations is represented by color intensity, with red indicating a positive correlation and blue indicating a negative correlation. **(C)** Differences in immune cell expression between high-risk and low-risk groups are considered statistically significant if *p* < 0.05. **(D)** The correlation between risk genes and risk scores, which is essential for model construction and immune cell analysis, is illustrated using color intensity: red denotes a positive correlation, whereas blue denotes a negative correlation. **(E)** The variations in Tumor Microenvironment (TME) scores, encompassing ESTIMATEScore, ImmuneScore, and StromalScore, between high-risk and low-risk groups are analyzed to evaluate differences in the tumor microenvironment. **(F)** The drug sensitivity analysis indicates that certain drugs exhibit increased efficacy in high-risk groups. **(G)** Conversely, the drug sensitivity analysis identifies drugs that are particularly effective in low-risk groups. Statistical significance is denoted as follows: *p < 0.05, **p < 0.01, and ***p < 0.001.

The immune system is vital in the onset of illnesses. We are currently investigating the relationship between efferocytosis and immunity. Initially, we adopted the CIBERSORT method to evaluate the prevalence of immune cells. Upon comparison, we discovered a significant correlation between the content of immune cells and risk scores. The distribution of immune cell content varies among different risk groups, and each sample also exhibits differences in immune cell content and type (Figure 6A). In Figure 6B, the relationship between immune cells is illustrated, with red indicating a positive correlation and blue indicating a negative one. A deeper hue indicates a more significant correlation. Out of the identified immune cells, 15 are significantly correlated with the risk score, with four showing a positive correlation and the remainder showing a negative correlation (Supplementary Figure S4). Analysis of the differential expression of these immune cells in high- and low-risk groups revealed that 16 immune cells had varying expression levels (Figure 6C). Further analysis showed correlations between IL33, SIRPG, and most immune cells, as well as the risk scores and five genes involved in the model construction (Figure 6D). After conducting an immune correlation analysis on the risk model, we proceeded to investigate the TME in the high- and low-risk categories. Our analysis revealed that the low-risk cohort demonstrated elevated TME scores in StromalScore, ImmuneScore, and ESTIMATEScore (Figure 6E). These results point to significant differences in the immune microenvironment among subgroups identified based on efferocytosis-related risk scores. This may explain why BRCA patients with different risk levels experience varying immunotherapy effects, providing a basis for personalized immunotherapy and patient-specific treatment targets.

### 3.6 Screening potential therapeutic drugs

The analysis of the relationship between the drug’s IC50 value and the risk score revealed 105 drugs with notable variations in IC50 values across the two groups. In particular, 98 medications demonstrated significant sensitivity in the high-risk category, whereas seven medications showed notable sensitivity in the low-risk category. Figure 6F showcased four medications that exhibited significant sensitivity in the high-risk cohort, indicating their possible efficacy for treating these patients. On the other hand, Figure 6G showcased four drugs with high sensitivity in the low-risk group, indicating their potential therapeutic benefits for low-risk patients. These findings provided diverse drug options for BRCA patients at different stages, aiming to enhance drug effectiveness and utilization, and ultimately alleviate patients’ symptoms. For more details on the drugs, please refer to Supplementary Table 4.

### 3.7 Screening for genes with independent prognostic value

**FIGURE 7:**
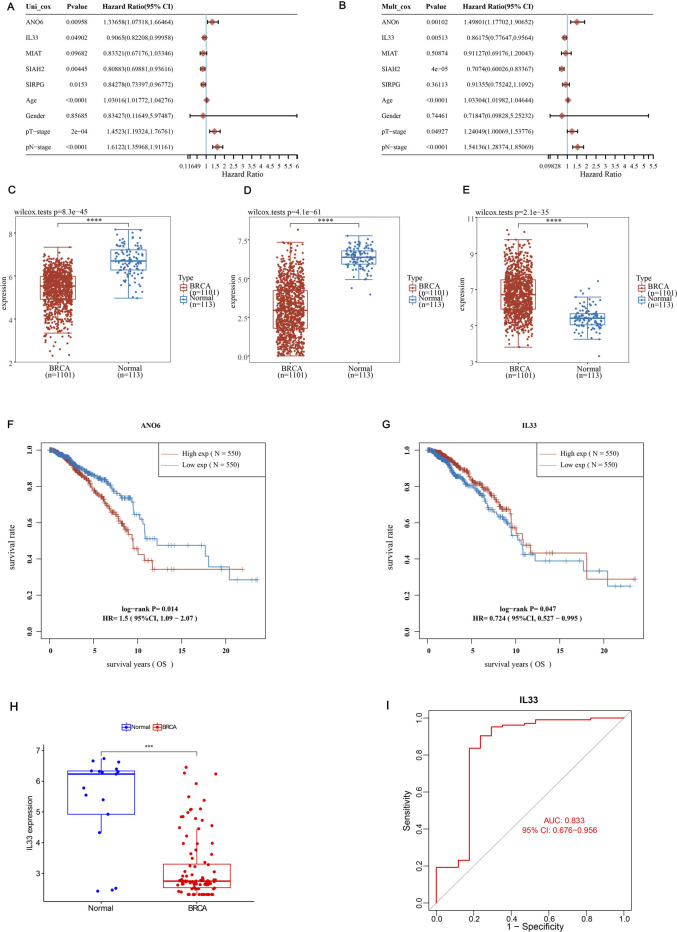
Screening for independent prognostic genes. **(A, B)** Hazard ratios and *p*-values for the components in BRCA were estimated utilizing univariate and multivariate Cox regression models, which integrated both clinical parameters and five prognostic ERGs. **(C–E)** The expression levels of ANO6, SIAH2, and IL33 were examined in BRCA tissues in comparison to adjacent normal tissues. **(F, G)** The overall survival (OS) of BRCA patients exhibiting high *versus* low expression levels of ANO6 and IL33 was compared. **(H)** The expression level of IL33 in normal tissues and BRCA was verified using the GSE42568 dataset. **(I)** The ROC curve of IL33 was constructed using the GSE42568 dataset to study the contribution of IL33 to the model. Statistical significance was determined with thresholds set at *p < 0.05, **p < 0.01, and ***p < 0.001.

In our research investigating the impact of ERGs on BRCA, we adopted both univariate and multivariate Cox regression analyses to assess the *p*-values, HRs, and 95% CIs for five significant genes, along with clinical features incorporated into our model. The analysis identified ANO6, IL33, and SIAH2, along with age, pT stage, and pN stage, as independent prognostic factors for BRCA (Figures 7A, B; *p* < 0.05). Our analysis of differential expression indicated that ANO6 and IL33 were markedly downregulated in cancerous tissues relative to normal ones (Figures 7C, D), whereas SIAH2 showed elevated expression in tumor samples (Figure 7E). Finally, we compared the survival expression of these three genes and found that except for SIAH2, the other two had survival differences (Supplementary Figure S5). Among them, the survival rate of the ANO6 high expression group was significantly lower than that of the low expression group, while the survival rate of the IL33 high expression group was significantly higher than that of the low expression group (Figures 7F, G). Specifically, high expression of ANO6 correlated with poorer survival, while high expression of IL33 was associated with better survival. Given the adverse survival outcomes linked to low IL33 expression in BRCA, we chose to focus on IL33 for further investigation. Then, we screened GSE42568 (including 104 BRCA samples and 17 normal samples) from the GEO database to verify the expression of IL33 and construct the ROC curve. The results showed that IL33 was lowly expressed in BRCA (Figure 7H), and the ROC curve showed an AUC value of 0.833 (Figure 7I), indicating that IL33 had a high contribution to the constructed model.

### 3.8 Assessment of IL33 levels and prognostic role in BRCA

**FIGURE 8:**
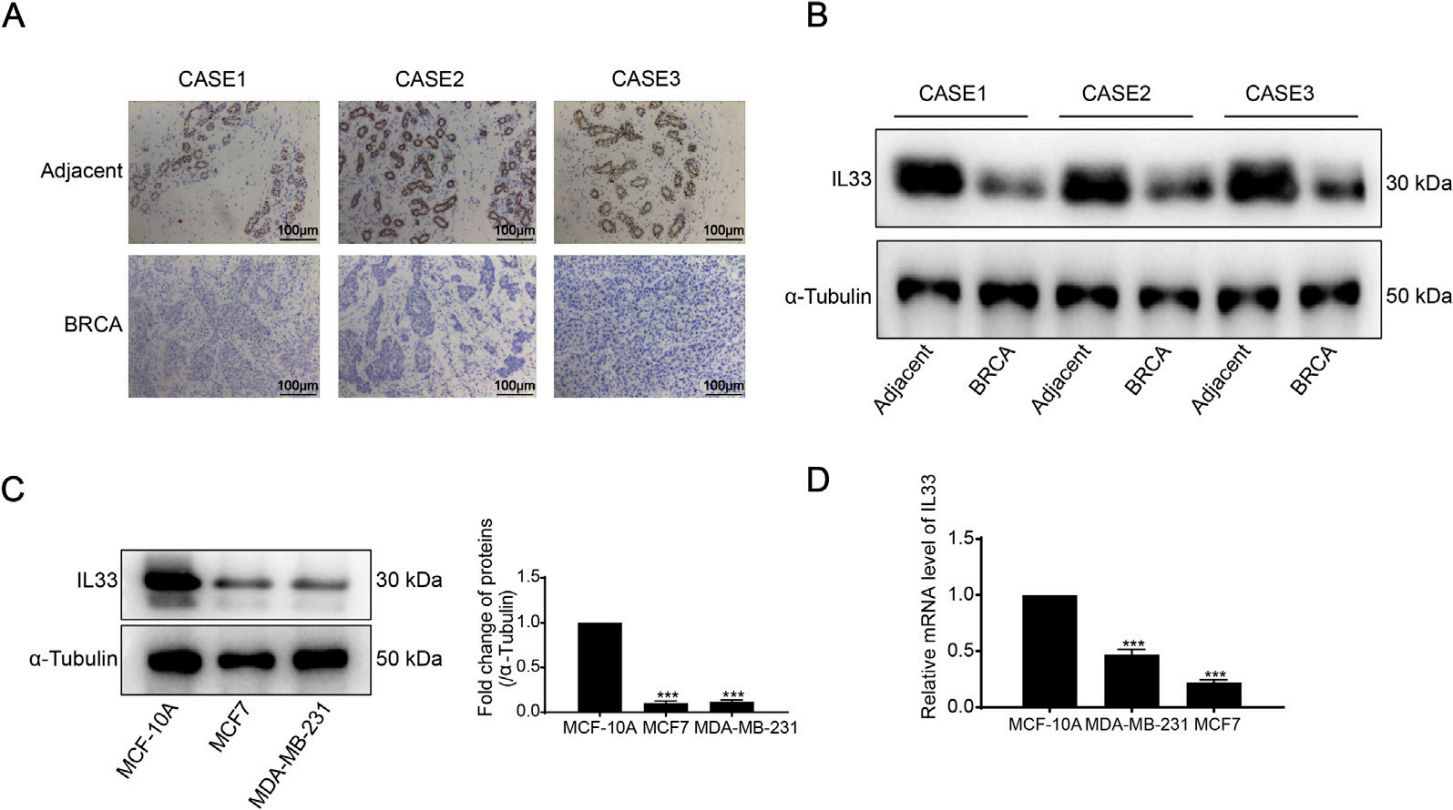
**(A)** Immunohistochemistry (IHC) was employed to detect IL33 expression in BRCA tissues and adjacent normal tissues. **(B)** IL33 expression in BRCA and adjacent tissues was evaluated using Western blot (WB) analysis. **(C)** The expression of IL33 in two BRCA cells and one normal cell line was determined by WB. **(D)** chain reaction (PCR) was also utilized to assess IL33 expression in the same cell lines. The scale for plotting was set at 100 μm.

To evaluate IL33 expression in BRCA, we conducted IL33 staining on three adjacent tissue samples and three BRCA tissue samples. Immunohistochemistry (IHC) and Western blot (WB) analyses revealed significantly lower levels of IL33 in BRCA tissues compared to adjacent tissues (Figures 8A, B). We evaluated IL33 levels in two breast cancer cell lines and a normal cell line through WB and PCR techniques. The BRCA cell lines exhibited a significant decrease in both IL33 protein and mRNA levels when compared to the normal cell line (Figures 8C, D).

**FIGURE 9:**
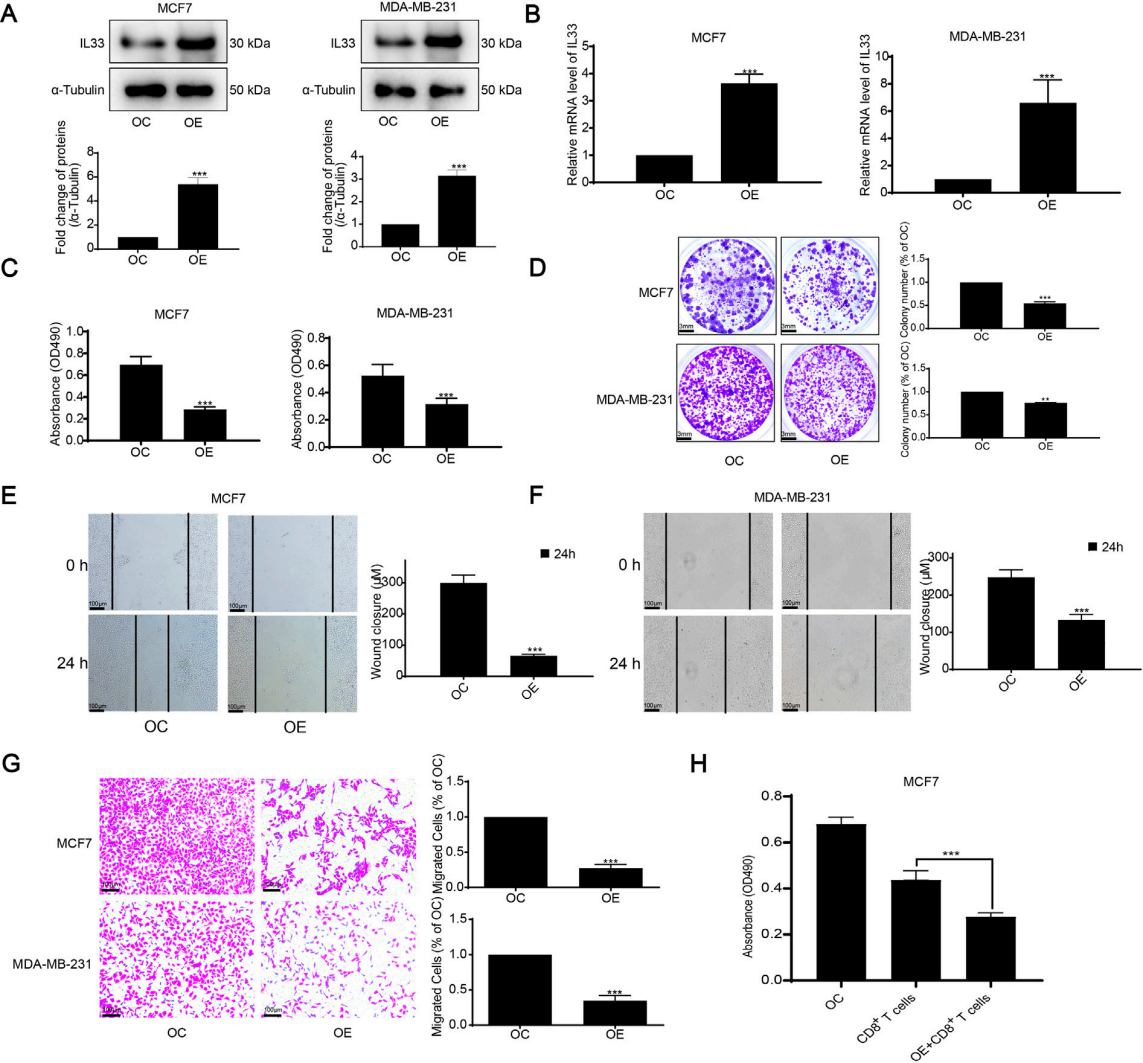
Assessment of IL33 prognostic role in BRCA. **(A)** We assessed IL33 expression using WB following its overexpression. **(B)** PCR was employed to measure IL33 expression in tumor cells following its overexpression. **(C)** Cell proliferation was assessed by using the MTT assay following IL33 overexpression in tumor cells. **(D)** The clonogenic assay was employed to assess colony formation suppression following IL33 overexpression in tumor cells. **(E–G)** We utilized scratch and transwell assays to assess the suppression of migration in tumor cells following IL33 overexpression. **(H)** We assessed the survival of OC and OE tumor cells following treatment with CD8^+^ T cells. Statistical significance was determined with thresholds set at *p < 0.05, **p < 0.01, and ***p < 0.001.

Furthermore, considering the role of IL33 in BRCA, we validated its overexpression (OE) through WB and PCR techniques (Figures 9A, B). Subsequently, we assessed the effects of IL33 on BRCA using MTT, clonogenic, scratch, and transwell tests. Overexpression of IL33 significantly inhibited cell proliferation, clonogenicity, and migration (Figures 9C–G). Furthermore, co-incubation of human BRCA cells with CD8^+^ T cells demonstrated that activated T cells were more effective at eliminating cancer cells in the presence of overexpressed IL33 (Figure 9H). These results underscore IL33’s significant role in BRCA cell proliferation, metastasis, and immune evasion.

### 3.9 Increased IL33 levels suppressed the proliferation and movement of BRCA cells in a living organism

**FIGURE 10:**
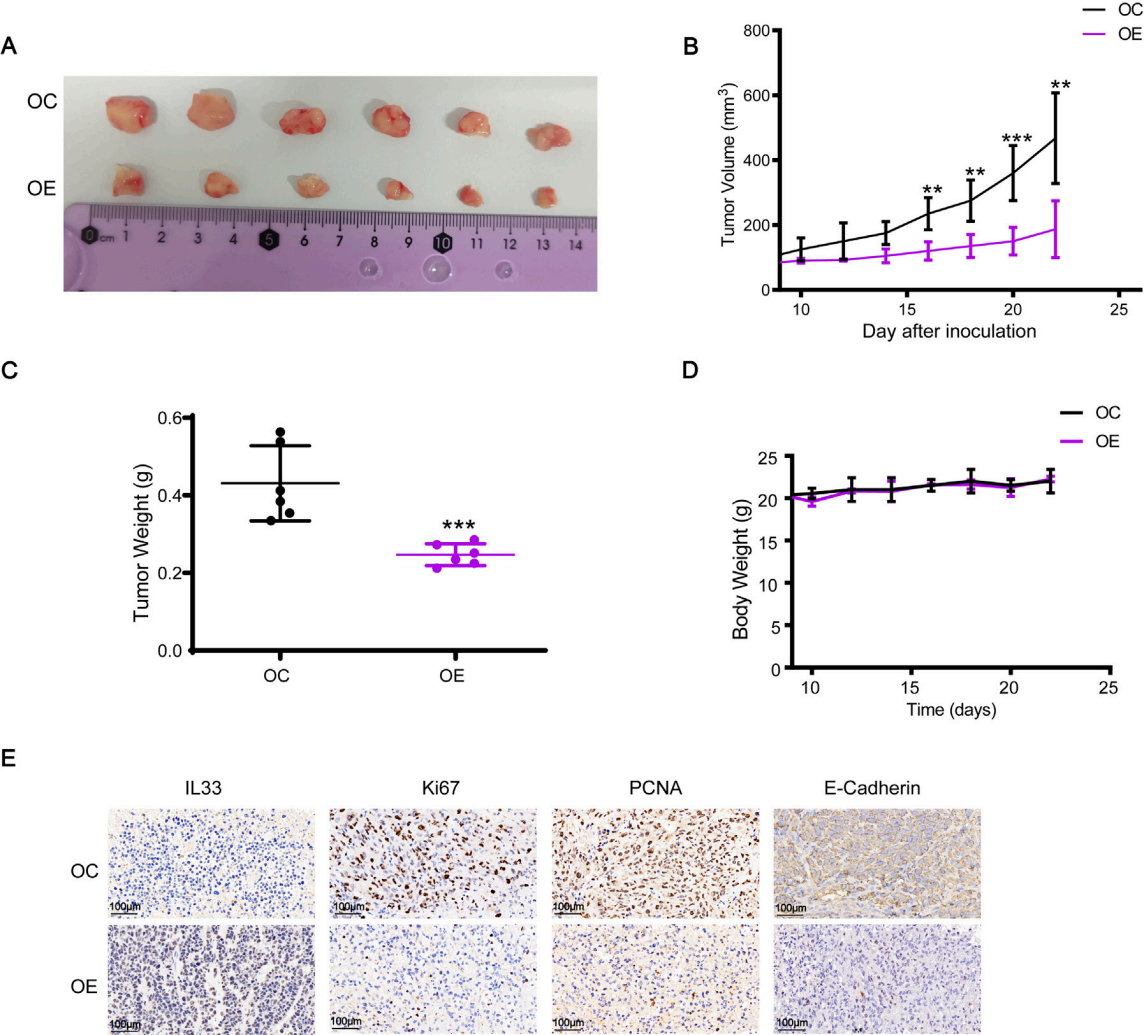
IL33 overexpression inhibited the growth and migration of BRCA cells *in vivo*. **(A, B)** Cell suspensions of 2 × 10^6^ MDA-MB-231-OC and MDA-MB-231-OE were injected into the right flank of mice. Tumor volume was measured every 2 days once it reached 70–100 mm³, approximately 8 days after injection. After 22 days, the tumors were excised and photographed. **(C)** The tumor weight was statistically analyzed at 22 days post-injection to evaluate differences between the MDA-MB-231-OC and MDA-MB-231-OE groups. **(D)** Changes in the body weight of each group of mice were monitored and recorded throughout the experiment to assess the effects of IL33 overexpression on overall health and to ensure that any observed tumor-related effects were not confounded by significant weight loss. **(E)** Immunohistochemistry was employed to detect the expression levels of IL33, Ki67, PCNA, and E-Cadherin in tumor tissues. The images were captured with a scale of 100 μm.

We also examined the influence of IL33 overexpression on tumor development *in vivo* using a subcutaneous xenograft model in nude mice. Mice were injected with cells that overproduce IL33 (OE) and with control cells (OC). Results showed a significant reduction in tumor growth in the OE group compared to the OC group (Figures 10A, B). Furthermore, the tumors in the OE group were significantly less heavy compared to those in the OC group, as shown in Figure 10C. Importantly, the average body weight of the mice remained stable regardless of IL33 overexpression (Figure 10D). The immunohistochemical examination of proliferation indicators Ki-67 and PCNA showed a significant reduction in cells positive for Ki-67 and PCNA in the OE group compared to the OC group (Figure 10E). Moreover, IL33 overexpression led to reduced levels of the migration-related protein E-Cadherin *in vivo* (Figure 10E). These results strongly indicate that IL33 overexpression impedes BRCA cell growth and migration *in vivo* by downregulating Ki-67, PCNA, and E-Cadherin.

## 4 Discussion

Efferocytosis is a mechanism where the body removes dying cells to preserve tissue balance. Irregular cytosis may lead to a range of illnesses, including heart conditions, metabolic disorders, neurodegenerative diseases, and cancer (Doran et al., 2020). Therefore, it is crucial to improve therapeutic strategies and discover potential therapeutic mechanisms by improving efferocytosis. In recent years, research on efferocytosis in cancer has made great progress. Research indicates that molecules and pathways involved in efferocytosis are intimately linked to cancer progression, metastasis, and treatment resistance. Efferocytosis also creates an immunosuppressive environment within the tumor and enables cancer cells to evade immune surveillance (Qiu et al., 2023). The process of efferocytosis mainly includes three stages: intracytoplasmic cell sensing and chemotactic migration to apoptotic cells (AC), intracellular cell recognition of AC, and phagocytosis and digestion of AC (Boada-Romero et al., 2020). When a process occurs abnormally, it can lead to disease. Efferocytosis may also trigger the creation of a tumor microenvironment that suppresses the immune system, resulting in reduced immune activity. This can cause tumor cells to grow uncontrollably and speed up the development of the disease (Qiu et al., 2023). Efferocytosis significantly contributes to the resistance observed in cancer therapies. Gene groups linked to efferocytosis, including Tyro3, Axl, MerTK, Gas6, BAI-1, CX3CL1, CD31, CD47, Rac1, and the TIM family, represent significant targets for prospective cancer therapies. Changes in these processes can lead to drug resistance and diminish the effectiveness of cancer therapy (Zhou et al., 2020; Werfel and Cook, 2018; Cheng et al., 2022). However, there are few studies on the role of efferocytosis in BRCA. Our research involved a comprehensive bioinformatics examination of ERGs in BRCA, and we identified the significance of specific genes for the first time. This provides a valuable reference for future research.

In this study, researchers attempted to establish a prognostic model of ERGs based on BRCA. They included a total of five prognosis-related genes (ANO6, IL33, MIAT, SIAH2, SIRPG) in the model construction. Various studies have demonstrated that these genes are intimately linked to the development and progression of cancer. Anoctamin 6 (ANO6) is part of the anoctamin family, which consists of 10 proteins (ANO1-10). ANO6 plays a role in cell movement, regulation of cell size, and the exposure of phosphatidylserine on the cell surface. Studies suggest it serves as a prognostic indicator for BRCA, potentially by triggering stroma-associated pathways and encouraging macrophage polarization, thereby influencing BRCA development (Tang et al., 2023). However, further validation is needed. Interleukin-33 (IL-33), a member of the IL-1 family, is an alarmin cytokine that plays an important role in tissue homeostasis and repair, cancer, etc (Cayrol and Girard, 2022). IL33 is an important gene whose expression level is also related to the immune microenvironment. By targeting IL-33, the therapeutic effect of BRCA can be enhanced by regulating excessive immune responses, reducing inflammatory damage, or fighting tumor cells by enhancing immune responses (Donahue et al., 2024). In BRCA, studies have found that IL-33 is involved in the metastasis process of BRCA, mainly by changing the immune microenvironment of the type 2 inflammatory metastasis microenvironment (Shani et al., 2020) and promoting BRCA lung metastasis. This may be related to the involvement of IL33 in cell adhesion molecules cams, chemokine signaling pathways, etc., which jointly regulate the occurrence and development of BRCA. MIAT is a long non-coding RNA whose expression levels vary in different breast cancers. MIAT silencing can lead to tumor cell growth arrest, thereby increasing the sensitivity of BRCA treatment (Almnaseer and Mourtada-Maarabouni, 2018). SIAH2 can mediate ubiquitination and degradation of substrates and regulate multiple signaling pathways in response to hypoxic stress, thereby promoting tumor occurrence and progression (Chan et al., 2017; Liu et al., 2022). SIRPG is upregulated in human lung adenocarcinoma, and its overexpression predicts poor survival outcomes (Xu et al., 2022). In BRCA, SIRPG also has good predictive performance and is beneficial for the treatment of BRCA (Xing et al., 2023).

Determining the immune profile of the TME is crucial for immunotherapy (Gajewski et al., 2017). Our study analyzed immune expression levels across different risk categories, revealing variations between groups at high and low risk. We also observed variations in the distribution of different immune cells, suggesting that patients with different risks may exhibit different symptoms. Tailoring treatment based on individual needs can lead to more effective treatment plans. Research has shown that the infiltration patterns of immune cells contribute to the complexity and variety of the TME. This involvement can help regulate the biological processes, clinical outcomes, genetic variation, and more in tumors, ultimately benefiting the precise immunotherapy of those with cancer (Xu et al., 2021). Moreover, there is an inseparable relationship between immune processes and treatment resistance. By enhancing the patient’s immune function, the patient’s drug resistance can be effectively reduced and the patient’s therapeutic effect can be improved (Chang et al., 2023). In our study, we found that there were differences in the sensitivity of 105 drugs between the high-risk group and the low-risk group. This may be related to the biological characteristics, immune microenvironment, gene mutation status, etc., of different patients. Therefore, it is of great reference value to select more sensitive drugs for different populations to achieve personalized treatment. In recent years, immunotherapy has been highly successful in treating BRCA. Dendritic cells are adaptable antigen-presenting cells essential for starting and managing both innate and adaptive immune reactions. Research on the impact of dendritic cell vaccines is essential for understanding immune regulation and other aspects of BRCA (Qian et al., 2023). Consequently, identifying and developing specific markers is crucial for comprehending BRCA subtypes and selecting precise treatment strategies. IL33 can be a therapeutic target for BRCA and has strong research prospects. However, there are still many challenges. First, IL33 has a strong immune effect in tumor response, but in some cases, IL33 can also inhibit immune escape, which makes IL33 a strategy for targeted therapy complicated (Ryan et al., 2020). Second, the research mechanism of IL33 in tumors needs to be further explored to show its practical application. In the future, we will continue to explore the role of IL33 in BRCA to provide more evidence that IL33 can become a therapeutic target for BRCA.

In our research, we examined the prognostic significance and expression profile of ERGs in BRCA for the first time. Nevertheless, there are certain constraints in our research. Firstly, we only analyzed the general categories of BRCA, but we lacked prognostic validation for different types of BRCA, such as triple-negative breast cancer, which possess unique characteristics. Secondly, our study primarily relied on public databases, which lacked clinical patient data from specific medical institutions and did not represent local characteristics. Lastly, we only performed basic *in vivo* and *in vitro* validation of important genes, and further fundamental experiments are necessary to enhance our research.

## 5 Conclusion

This study highlights the critical role of ERGs in the progression of BRCA and identifies key prognostic markers. Our research demonstrates that a prognostic model based on ERGs effectively predicts survival outcomes and immune responses in BRCA patients. IL33 stands out as an important indicator, with both live and laboratory tests verifying its significance in the advancement of BRCA. This research, for the first time, clarifies the function of efferocytosis in BRCA and provides new perspectives for possible treatment approaches.

## Data Availability

The original contributions presented in the study are included in the article/Supplementary Material, further inquiries can be directed to the corresponding authors.
