# Supplementary material for: Efferocytosis-related gene IL33 predicts prognosis and immune response and mediates proliferation and migration in vitro and in vivo of breast cancer
Source: Front Pharmacol. 2025 Jan 22;16:1533571. doi: 10.3389/fphar.2025.1533571 (PMC11794308; doi:10.3389/fphar.2025.1533571)
Supplement: Supplementary file 1 [file DataSheet1.docx]

Supplementary Material

**Supplementary Figures 1-5 and Supplemental Tables 1-4**

**Supplementary Figures 1-5**

**Figure S1. GO enrichment analysis of 32 DEGs.**

**
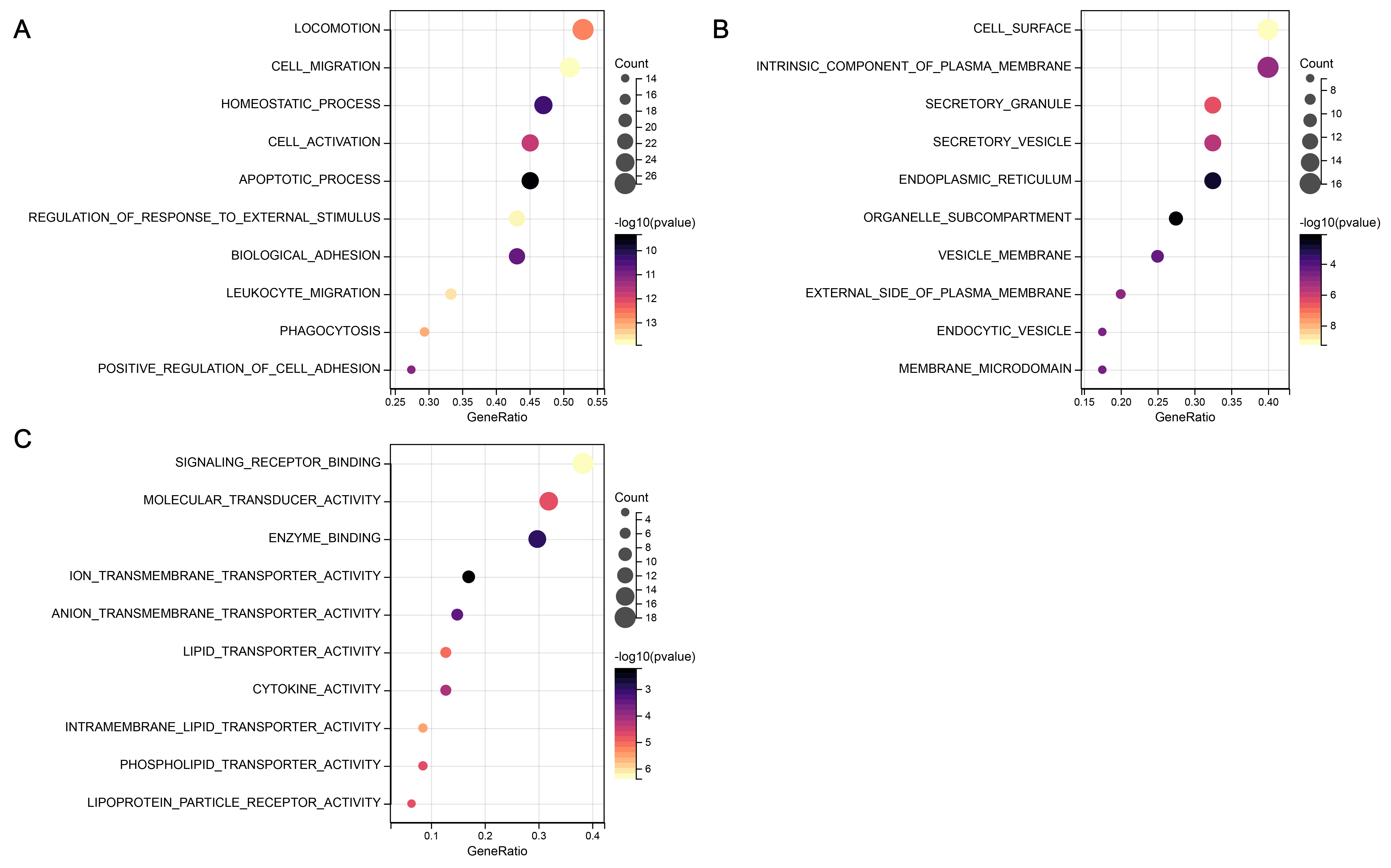
**

Figure S1. GO enrichment analysis of 32 DEGs. GO includes three parts: BP (Biological Process), CC (Cellular Component), and MF (Molecular Function). (A) Enrichment of DEGs in BP. (B) Enrichment of DEGs in CC. (C) Enrichment of DEGs in MF.

**Figure S2.**

**
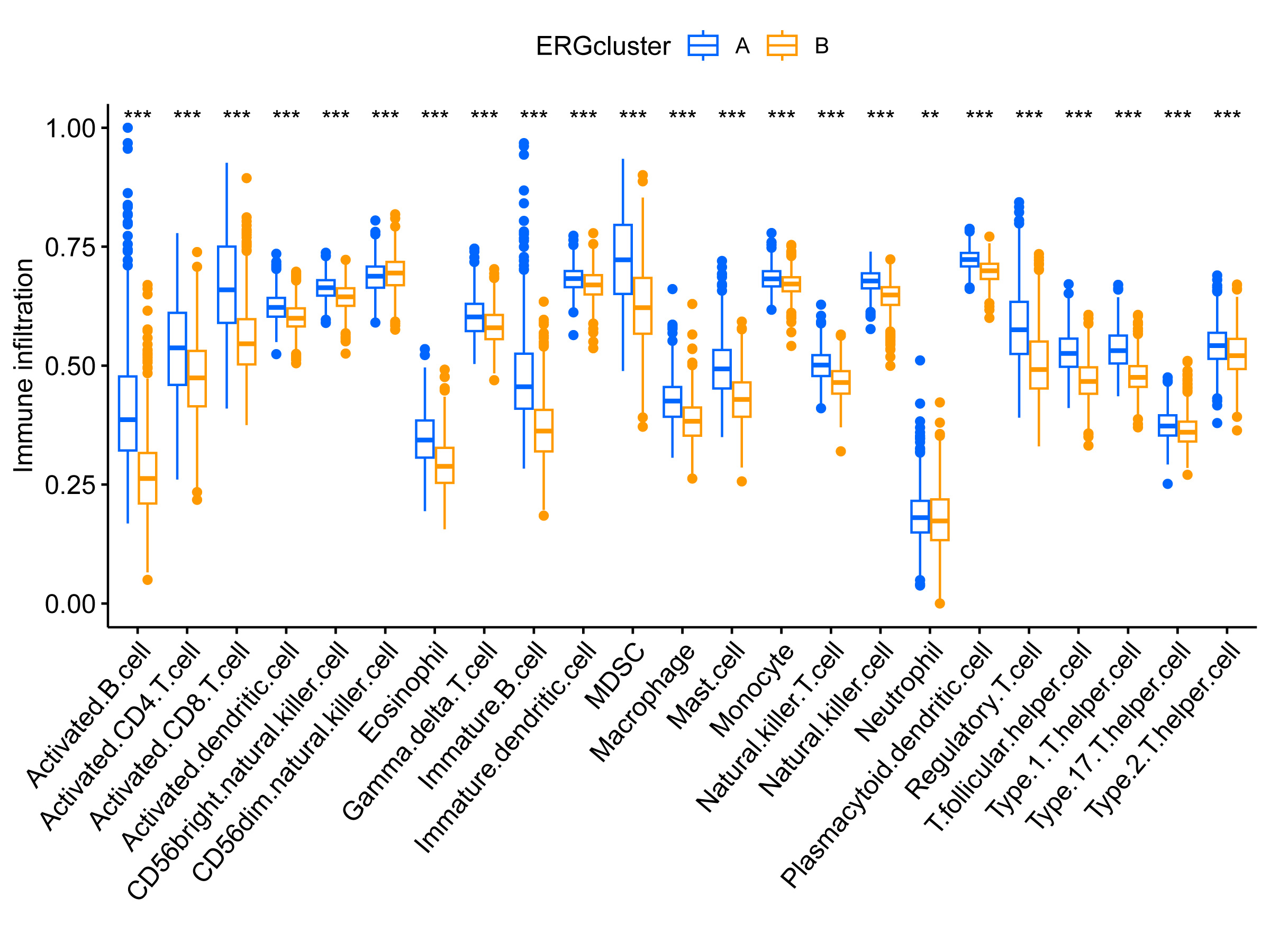
**

**Figure S2.** Level of immune infiltration of immune cells between two clustered subgroups.

**Figure S3.**

**
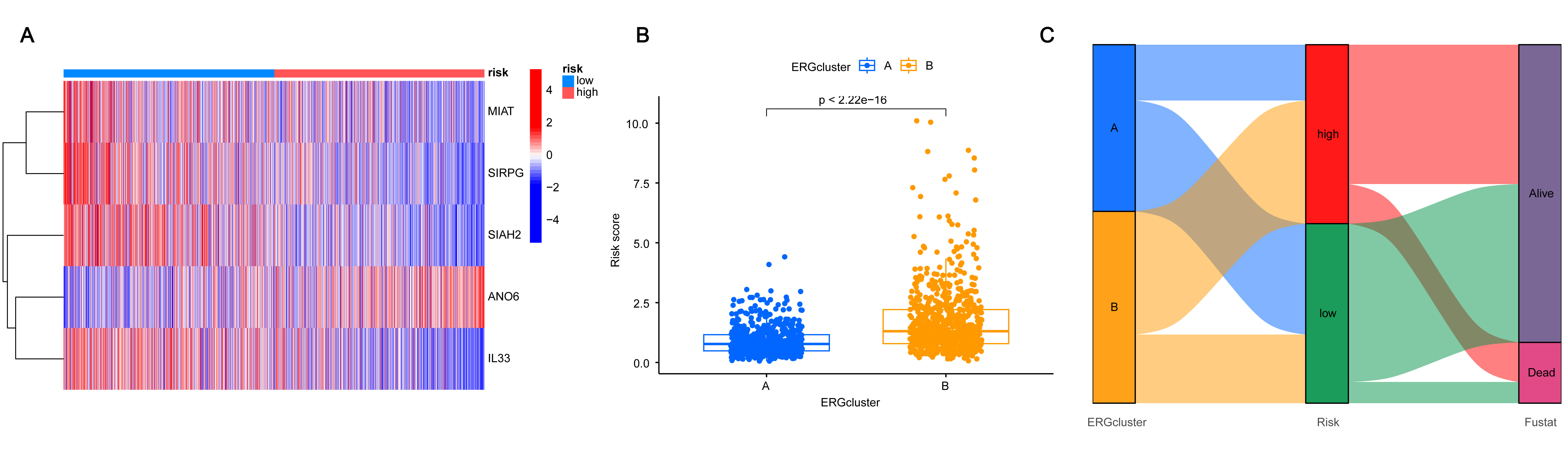
**

**Figure S32.** The expression differences of the five ERGs involved in model construction and the development trend of risk scores in the two cluster subgroups. (A) The heat map shows the expression differences of the 5 ERGs involved in building the risk model in high and low risk groups. (B) Expression differences of risk scores in two cluster subgroups. (C) Sankey diagram shows the risk trend and survival status trend of BRCA patients in two subgroups.

**Figure S4.**

**
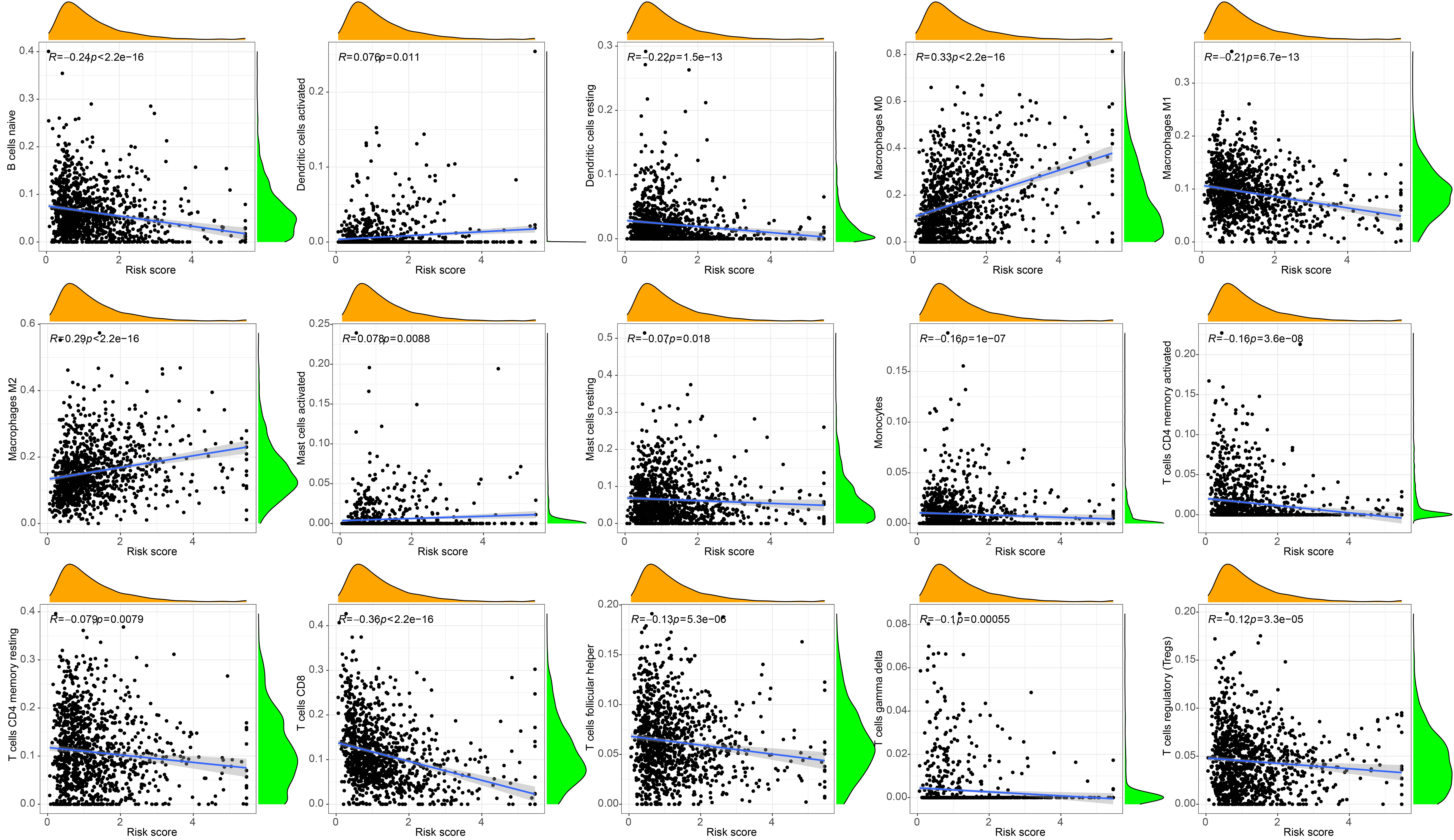
**

**Figure S4.** Correlation between immune cells and risk scores.

**Figure S5**

**
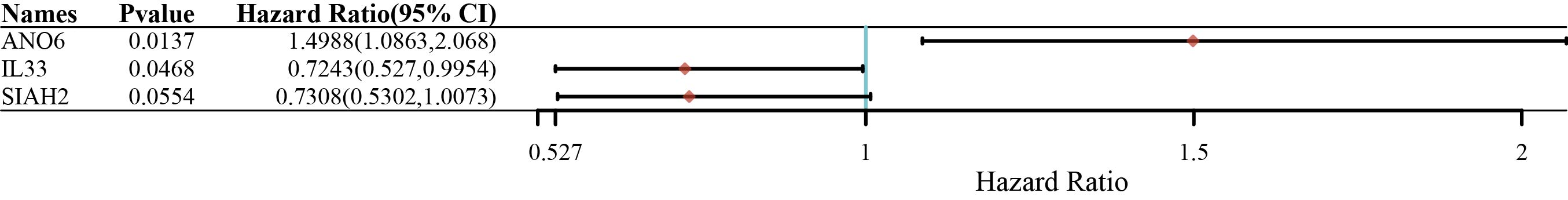
**

**Figure S5**. Forest plot showing *p*-values, HR (95% CI) for ANO6, SIAH2, and IL33.

**Supplementary Tables 1-4**

**Table S1.** The clinical characteristics of BRCA patients in the TCGA cohort.

| Clinical characters | Number |
| --- | --- |
| Gender  Male  Female | 12  1089 |
| Age |  |
| Mean (SD) | 58.4 (13.2) |
| Median [MIN, MAX] | 58 [26,90] |
| TNM stage  I  II  III  IV  X | 182  624  251  20  13 |
| pT_stage  T1  T2  T3  T4  TX | 281  639  138  40  3 |
| pN_stage  N0  N1  N2  N3  NX | 516  366  120  79  20 |
| pM_stage  M0  M1  MX | 910  22  163 |

**Table S2.** 272 genes were downloaded from the Genecards database and Harmonizome database.

| Gene |
| --- |
| ABCA1 |
| ABCC11 |
| ABCG1 |
| ACKR2 |
| ADAM10 |
| ADAM17 |
| ADAM9 |
| ADGRB1 |
| AGER |
| ALDH2 |
| ALOX12 |
| ALOX15 |
| ALOX15B |
| ALOX5 |
| ANG |
| ANO3 |
| ANO4 |
| ANO5 |
| ANO6 |
| ANO7 |
| ANO9 |
| ANXA1 |
| ANXA2 |
| APOE |
| ARG1 |
| ARG2 |
| ARHGEF33 |
| ARNT |
| ATP11A |
| ATP11B |
| ATP11C |
| ATP2A1 |
| ATP2A2 |
| ATP2A3 |
| ATP8A1 |
| ATP8A2 |
| AXL |
| BCAR1 |
| BSG |
| C1QA |
| C1QB |
| C1QC |
| C3 |
| CALR |
| CAMK2A |
| CAMK2B |
| CAMK2D |
| CAMK2G |
| CASP1 |
| CASP3 |
| CASP7 |
| CD14 |
| CD24 |
| CD274 |
| CD300LF |
| CD36 |
| CD47 |
| CD5L |
| CEBPB |
| CERNA3 |
| CH25H |
| CLU |
| CPT1A |
| CPT1B |
| CPT1C |
| CREB1 |
| CRK |
| CRKL |
| CX3CL1 |
| CX3CR1 |
| DNMT3A |
| DOCK1 |
| DUSP16 |
| DUSP2 |
| DUSP4 |
| DUSP5 |
| DUSP7 |
| DUSP8 |
| DYNLT1 |
| EDIL3 |
| EGLN3 |
| ELANE |
| ELMO1 |
| EPO |
| EPOR |
| FGL2 |
| FN1 |
| FPR2 |
| FPR3 |
| GABARAP |
| GAPDH |
| GAS5 |
| GAS6 |
| GATA2 |
| GPR101 |
| GPR132 |
| GPR18 |
| GULP1 |
| HAVCR1 |
| HAVCR2 |
| HIF1A |
| HMGB1 |
| ID3 |
| IFNB1 |
| IGF2R |
| IL10 |
| IL1B |
| IL1RN |
| IL33 |
| IL6 |
| IL6R |
| IRF3 |
| ITGAV |
| ITGB3 |
| ITGB5 |
| JAK2 |
| KNG1 |
| LGALS3 |
| LGR6 |
| LINC01150 |
| LINC01587 |
| LINC01672 |
| LINC02605 |
| LIPA |
| LOC106694316 |
| LRP1 |
| MAP2K1 |
| MAP2K2 |
| MAPK1 |
| MAPK11 |
| MAPK12 |
| MAPK13 |
| MAPK14 |
| MAPK3 |
| MAPKAPK2 |
| MBL2 |
| MBL3P |
| MEG8 |
| MEGF10 |
| MEGF11 |
| MERTK |
| MFGE8 |
| MIAT |
| MIR125A |
| MIR126 |
| MIR1293 |
| MIR148B |
| MIR190B |
| MIR21 |
| MIR216A |
| MIR33A |
| MIR33B |
| MIR34A |
| MIR379 |
| MIR409 |
| MIR7-3HG |
| MIRLET7C |
| MIRLET7D |
| MPO |
| MSR1 |
| MTOR |
| NCF1 |
| NFATC1 |
| NFATC2 |
| NFATC3 |
| NFATC4 |
| NFE2L2 |
| NLRP3 |
| NR1H2 |
| NR1H3 |
| NRF1 |
| NRP2 |
| NTN1 |
| ODC1 |
| P2RY12 |
| P2RY2 |
| P2RY6 |
| PANX1 |
| PBX1 |
| PECAM1 |
| PGR-AS1 |
| PHACTR1 |
| PLA2G15 |
| PLA2G6 |
| PLAUR |
| PLCG1 |
| PLG |
| PLGRKT |
| PPARD |
| PPARG |
| PROS1 |
| PTGER2 |
| PTGER4 |
| PTGS2 |
| PTK2 |
| PTPN11 |
| PTPN6 |
| PWAR1 |
| PWAR4 |
| PWAR6 |
| QPCTL |
| RAB14 |
| RAB17 |
| RAB4B-EGLN2 |
| RAB5A |
| RAB5B |
| RAB5C |
| RAB7A |
| RAB7B |
| RAC1 |
| RHOA |
| RMRP |
| RXRA |
| S1PR1 |
| S1PR5 |
| SCARB1 |
| SCARF1 |
| SERPINA1 |
| SFTPD |
| SGK1 |
| SIAH2 |
| SIGLEC10 |
| SIRPA |
| SIRPB1 |
| SIRPG |
| SIRT1 |
| SIRT6 |
| SLC16A1 |
| SLC2A1 |
| SLC66A1 |
| SMAD5-AS1 |
| SMILR |
| SNHG14 |
| SOD2-OT1 |
| SPHK1 |
| SPHK2 |
| ST2 |
| STAB1 |
| STAB2 |
| TGFB1 |
| TGFB3 |
| TGFBRAP1 |
| TGM2 |
| THBS1 |
| TIMD4 |
| TLR3 |
| TLR9 |
| TMEM256-PLSCR3 |
| TMEM30A |
| TNFSF13B |
| TRA-TGC7-1 |
| TREM2 |
| TRE-TTC3-1 |
| TYRO3 |
| UBE2D3 |
| UCP2 |
| UQCRFS1 |
| VPS11 |
| VPS16 |
| VPS18 |
| VPS33A |
| VPS39 |
| VPS41 |
| VPS8 |
| VTN |
| WDFY3 |
| XKR4 |
| XKR6 |
| XKR7 |
| XKR8 |
| XKR9 |
| XRCC4 |

**Table S3.** Five ERGs were selected by multivariate Cox results.

| ERGs | Coef |
| --- | --- |
| ANO6 | 0.637634391712056 |
| IL33 | -0.220865917940123 |
| MIAT | -0.223869462386534 |
| SIAH2 | -0.496867864577252 |
| SIRPG | -0.224643319313097 |

ERGs, efferocytosis-related genes; Coef, coefficient.

**Table S4.** Detailed IC50-values for the 105 drugs.
